# β-blockers after myocardial infarction and 1-year clinical outcome – a retrospective study

**DOI:** 10.1186/s12872-020-01441-0

**Published:** 2020-04-09

**Authors:** Tora Hagsund, Sven-Erik Olsson, J. Gustav Smith, Bjarne Madsen Hardig, Henrik Wagner

**Affiliations:** 1 Medical faculty, Lund University, 22242 Lund, Sweden; 2 Department of Cardiology, Helsingborg Hospital, 251 87 Helsingborg, Sweden; 3 Department of Cardiology, Lund University, 22242 Lund, Sweden

**Keywords:** Beta-blockers, Myocardial infarction, Secondary prevention, Riks-HIA

## Abstract

**Background:**

Long term β-blocker therapy after myocardial infarction (MI) reduces mortality and recurrent MI but evidence for this treatment predates contemporary acute coronary care. β-blocker treatment is a key quality of care indicator in the Swedish national quality register for acute coronary care, Riks-HIA. Between 2011 and 2015 a declining number of MI-patients discharged with a β-blocker from the coronary care unit (CCU) at Helsingborg and other hospitals was reported. This retrospective observational study aimed to investigate the causes for discharge without a β-blocker and relate it to outcome, compared to patients discharged with a β-blocker.

**Methods:**

MI-patients registered in Riks-HIA discharged without β-blocker during 2011–2015 (no-β-group) and a control group (β-group) comprised of patients discharged with β-blocker treatment between January 1 to December 31, 2013, were matched by RIKS-HIA criteria for β-blocker use. Clinical characteristics, date of death, readmission for MI, other cardiovascular events were collected from Riks-HIA and medical records.

**Results:**

The no-β-group included 141 patients, where 65.2% had a justified reason for non-β-blocker use. The β-group included 206 patients. There was no difference in cardiovascular risk factor profile. There were a trend towards a higher number of readmissions for MI in the no-β-group was (*n* = 8 (5.7%) vs *n* = 2 (1.0%), *p* = 0.02), but not mortality (6 (4.3%) vs 2 (1.0%), *p* = 0.07) and combined readmission for angina pectoris, heart failure, arrhythmias or stroke/TIA (*n* = 23 (16.3%) vs *n* = 25 (12.1%), *p* = 0.27).

**Conclusion:**

A majority of the patients in the no-β-group had a justified absence of a β-blocker. β-blocker treatment post-MI showed a trend towards fewer readmissions for MI. But important quality information is lacking to make a firm conclusion of the effect on outcome.

## Background

In the early 1980s, the importance of β-blockers as long-term treatment after Myocardial infarction (MI) was documented in randomized trials [1]. No randomized controlled trials (RCTs) of β-blockers post-MI have however been conducted in the setting of modern coronary care, i.e. after the introduction of statin treatment, wider use of percutaneous intervention (PCI), and the more efficient antiplatelet drugs such as ADP-receptor blockers [2–4]. Observational studies examining the effect of post-MI β-blockers in the setting of a revascularized myocardium have however been conducted, with contradictory results [5–7]. A recent meta-analysis of observational studies of β-blocker treatment after acute MI, in patients who had undergone primary PCI, concluded that β-blockers post-MI was associated with lower 1-year all-cause mortality, but not a lower incidence of reinfarction or cardiac death [8].

According to current (2015) guidelines from European Society of Cardiology (ESC) for ST-Elevation Myocardial Infarction (STEMI) patients, long-term treatment with β-blockers is recommended to all patients without contraindications. However, this is a class II, level of evidence B recommendation, since contemporary RCTs are lacking [9]. According to American guidelines, continuation of treatment with β-blocker for 3 years is strongly recommended (Class I) for STEMI patients with normal left ventricular function. Continuation after 3 years in this patient group is considered optional (Class IIa or IIb) [10]. Regarding non-ST-Elevation Myocardial Infarction (NSTEMI) patients, the ESC guidelines recommends long-term β-blocker treatment in patients with an ejection fraction of < 40% [11].

In current (2015) treatment recommendation from the Swedish National board of Health and Welfare, long term treatment with a β-blocker post-MI is only recommended to patients with left ventricular systolic dysfunction (LVDF) [12]. However, The Swedish Society of Cardiology recommends β-blockers as long-term treatment in all patients with a history of acute coronary syndrome (ACS) and who are without contraindications [13]. These recommendations thus deviate from those of the ESC and the Swedish National board of Health and Welfare.

Annual reports from the Swedish national registry of cardiac intensive care (Riks-HIA) has shown that between 2011 and 2015, the coronary care unit (CCU) at Helsingborg hospital as many other hospitals experienced an unsatisfying number of patients receiving β-blocker treatment at discharge, taken into account the Riks-HIA quality of care indicator (Table 1) [14].

**Table 1:** The Riks-HIA quality index

Quality indicator	0.5 points (%)	1 point (%)
Reperfusion in STEM/LBBB	80	85
Reperfusion STEM/LBBB within recommended time (PCI within 90 min and thrombolysis within 30 min)	75	90
Coronary angiography planned or performed in NSTEMI	75	80
LMWH/heparin/fondaparinyx during the care episode or PCI performed within 24 h in NSTEMI	90	95
ASA/antiplatelet anticoagulant drugs on discharge following MI	90	95
P2Y12 blockers on discharge following MI	85	90
Beta-blockers on discharge following MI	85	90
Lipid-lowering drugs on discharge following MI	90	95
ACEI/ARB on discharge following MI	85	90

*ACEI* angiotensin-converting-enzyme inhibitor, *ARB* angiotensin receptor blocker, *ASA* acetylsalicylic acid, *LBBB* left bundle branch block, *LMWH* low-molecular-weight heparin, *MI* myocardial infarction, *NSTEMI* non-ST-elevation myocardial infarction, *PCI* percutaneous coronary intervention, *Riks-HIA* Register of information and knowledge about Swedish heart intensive care, *STEMI* ST-elevation myocardial infarction

This study therefore aimed to investigate the reasons why a growing number of patients were discharged without a β-blocker and to examine the clinical outcome of patients discharged from the CCU at Helsingborg’s hospital with or without β-blocker treatment post MI.

## Methods

The study population was selected from Riks-HIA, which has been used as a national quality registry for cardiac intensive care since 1995 and covers all the 73 CCUs in Sweden. The aim of the registry is to monitor and compare how well the CCUs adhere to the guidelines and implementation of new treatments. It also monitors and compares short- and long-term survival at the different CCUs [13]. Riks-HIA comprises over 100 variables and includes the majority of patients admitted to the CCUs of the participating hospitals. Two patient groups discharged from Helsingborg CCU were identified using the registry. The first patient group (no-β-group) comprised all MI patients who were discharged without a β-blocker, during 1 January 2011 to 1 January 2015. A control group (β-group) comprised all patients discharged with β-blocker treatment during 1st January 2013 to 31st December 2013. Patients eligible for β-blocker treatment was chosen based on Riks-HIA’s criteria for β-blocker treatment, (age < 80, discharged alive, absence of AV-block II or III and discharged with a diagnosis of a type 1 MI) [15].

In order to investigate similarities and differences between the no-β-group and β-group, relevant variables were selected and collected from the registries and from the patient’s medical records. All medical records were reviewed in order to verify the accuracy of the data collected from the registers, and to collect additional information. Regarding the no-β group, if a reason was stated why they did not receive a β-blocker and whether they were prescribed a β-blocker within a year from index event, this information was obtained. In the β-group, information was collected from the medical record regarding side effects attributable to β-blocker and whether treatment was terminated within a year.

Final diagnosis was determined using the WHO definition of type 1 MI [16]. In the medical record, this was identified by main diagnosis at discharge coded I21 in the international classification of disease (ICD) diagnostic tool, and subclass of MI (NSTEMI or STEMI) was obtained from the medical records.

The primary outcome was readmission for MI during 1 year after the index event. Secondary outcomes included all cause death, cardiovascular death or readmission for all cardiovascular events.

### Data processing and statistical calculations

IBM SPSS Statistics version 23 was used for all statistical calculations. Comparisons between the two groups were conducted using Fisher’s exact test for categorical variables, and Mann-Whitney U-test for continuous variables. The Bonferroni correction was applied for comparison of characteristics to adjust for multiple testing. Hence, a *p*-value of < 0.01 was considered significant after correction [17].

## Results

**Fig. 1:**
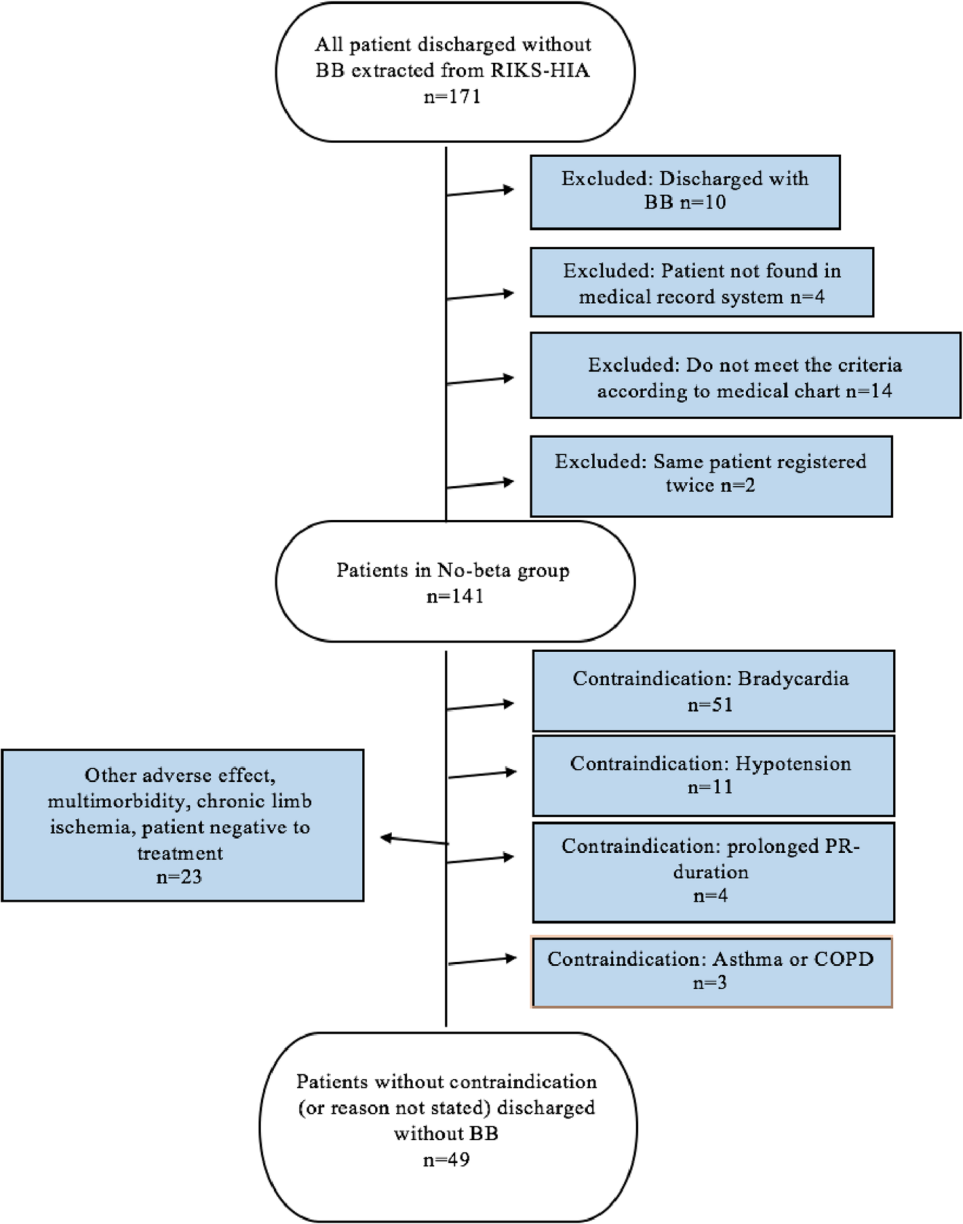
Flow-chart on patients who were admitted with a myocardial infarction to the coronary care unit at Helsingborg’s hospital between 1st January 2011 and 1st January 2015 and discharge without a β-blocker prescription. AV-block = Atrioventricular block, COPD = Chronic obstructive pulmonary disease, Riks-HIA = The Register of Information and Knowledge about Swedish heart intensive care

Data from Riks-HIA showed that of 1631 MI patients discharged from the CCU at Helsingborg hospital during the study period 1st of January 2011 to 1st of January 2015, 1155 patients met the criteria for β-blocker treatment. Among these 1155, a total of 171 patients (14.8%) were not prescribed a β-blocker. After reviewing the medical records, 24 of these patients (14.0%) turned out to be incorrectly registered as they did not meet the criteria for β-blocker treatment (*n* = 14) or were discharged with a β-blocker (*n* = 10). Consequently, 141 patients were included in the no-β-group (Fig. 1). The β-group included 206 patients. Among the 141 patients in the no-β-group, 92 patients (65.2%) had a reason stated in the medical record why they were discharged without and 49 patients (34.8%) were discharged without a β-blocker with no obvious reason (Fig. 1).

The two groups were similar to a large extent regarding risk factor profile (Table 2). There was a high prescription rate in both groups of the recommended secondary prevention related medications; statins, ASA and ADP receptor inhibitors. PCI was performed in 78.7% in the no-β-group, and in 82.5% in the β-group (Table 2). The baseline characteristics showed that mean heart rate and systolic blood pressure at admission was lower in the no-β-group (*p* = < 0.001 and *p* = 0.01) (Table 2). Ejection fraction < 50% at discharge was more common in the β-group (*p* = 0.01) (Table 2). Among medications at discharge, ACE inhibitors was to a wider extent prescribed in the β-group (*p* = < 0.001) (Table 2).

**Table 2:** Baseline characteristics in patients admitted with a type 1 myocardial infarction at the coronary care unit to Helsingborg’s hospital between 1 January 2011 and 1 January 2015 and discharged without a β-blocker prescription, compared to patients admitted with a type 1 myocardial infarction at the coronary care unit to Helsingborg’s hospital between 1 January 2013 and 31 December 2013 and discharged with a β-blocker prescription

	No-β-group *N* = 141 (Mean ± SD) or Mean (%)	β-group *N* = 206 (Mean ± SD) or Mean (%)	*P*-value
Background information, risk factors			
Age	64.7 ± 10.3	64.1 ± 10	0.57
Men	99 (70.2)	142 (68.9)	0.81
> 74 years	28 (19.9)	35 (17.0)	0.57
Smoker	40 (28.4)	78 (37.9)	0.08
Hypertension	59 (41.8)	108 (52.4)	0.06
Diabetes Mellitus	31 (22.0)	50 (24.3)	0.70
Heart failure	7 (5.0)	12 (5.8)	0.81
Chronic obstructive pulmonary disease	8 (5.7)	15 (7.3)	0.66
Asthma	9 (6.4)	9 (4.4)	0.46
Previous MI	19 (13.5)	39 (18.9)	0.19
Previous PCI	16 (11.3)	38 (18.4)	0.10
Previous CABG	12 (8.5)	22 (10.7)	0.58
Systolic blood pressure at admission ±SD	145.3 ± 24.3	152.4 ± 25.7	0.02
Heart rate at admission	71.6 ± 15.2	82.7 ± 19.3	< 0.001*
Diagnosis			
NSTEMI	89 (63.1)	120 (58.3)	0.37
STEMI	52 (36.9)	86 (41.7)	0.37
Coronary treatment during hospital stay			
PCI	111 (78.7)	170 (82.5)	0.40
CABG	3 (2.1)	10 (4.9)	0.25
Discharge			
Systolic blood pressure ± SD	134.2 ± 18.3	129.9 ± 18.1	0.02
Heart rate ± SD	67.2 ± 11.0^1^	67.9 ± 12.1^2^	0.88
Ejection fraction < 50%	28 (21.4)^3^	70 (35.4)^4^	0.01
Medical treatment at discharge			
ACE-I	73 (51.8)	149 (72.3)	< 0.001*
A2-I	20 14.2)	(15)	0.88
ADP-I	133 (94.3)	194 (94.2)	> 0.99
Statins	137 (97.2)	205 (99.5)	0.16
Aspirin	136 (96.5)	187 (90.8)	0.05
Oral anticoagulant	8 (5.7)	19 (9.2)	0.31
Long-acting nitrates	10 (7.1)	19 (9.2)	0.56
Calcium antagonists	33 (23.4)	29 (14.1)	0.03
Diuretics	20 (14.2)	40 19.4)	0.25

*Also significant after Bonferroni correction for mass significance: a *p*-value of < 0.02 was considered significant. ^1^Missing values *n* = 4, ^2^Missing values *n* = 2, ^3^Missing values *n* = 12, ^4^Missing values *n* = 8, *A2-I* Angiotensin II receptor antagonist, *ACE-I* Angiotensin converting enzyme inhibitor, *ADP-I* Adenosine diphosphate receptor inhibitor, *CABG* Coronary artery bypass grafting, *MI* Myocardial infarction, *NSTEMI* Non-ST-elevation myocardial infarction, *PCI* Percutaneous coronary intervention, *SD* Standard deviation, *STEMI* ST-elevation myocardial infarction

The non-β-group had a trend towards higher rate of readmissions for MI within 1 year after index event, compared with the β-group (Table 3). However, no statistically significant difference between the groups was found regarding other outcome parameters including all cause death, cardiovascular death, morbidity or readmission for all cardiovascular events (Table 3).

**Table 3:** Cardiovascular related readmissions and number of deceased patients during 1 year after index event in patients admitted with a type 1 myocardial infarction at the coronary care unit to Helsingborg’s hospital between 1 January 2011 and 1 January 2015 and discharged without a β-blocker prescription, compared to patients admitted with a type 1 myocardial infarction at the coronary care unit to Helsingborg’s hospital between 1 January 2013 and 31 December 2013 and discharged with a β-blocker prescription. First readmission counted only. TIA = Transient ischemic attack

	No-β-group *n* = 141 (Mean (%)	β-group *n* = 206 (Mean (%)	*p*-value
Readmission for myocardial infarction	8 (5.7%)	2 (1.0%)	0.02
Readmission for myocardial infarction, angina pectoris or heart failure	20 (14.2%)	17 (8.3%)	0.11
Readmission for myocardial infarction, angina pectoris, heart failure, arrhythmia, stroke or TIA	23 (16.3%)	25 (12.1%)	0.27
All cause death	6 (4.3%)	2 (1.0%)	0.07
Cardiovascular related death	1 (0.7%)	1 (0.5%)	> 0.99

*TIA* Transient ischemic attack

## Discussion

We studied the reasons to why β-blockers were not prescribed to all patients post-MI without an obvious contraindication at Helsingborg hospital during 2011–2015, and how this corresponded to clinical outcome. We found that, after reviewing the medical records, the treating cardiologist had motivated the absence of β-blocker in a majority of the patients. These motivations are however not visible in the registry and thus not presented in the annual reports from Riks-HIA.

When studying the correlation between lack of a β-blocker prescription and clinical outcome, our study showed that the patient group discharged without a β-blocker after MI had a significantly higher rate of readmissions for MI during 1 year after index event, compared with the patient group that was discharged with a β-blocker. However, no statistically significant difference between the patient groups was found regarding all cause death, cardiovascular death or readmission for all cardiovascular events, although a trend towards higher mortality without β-blockers was observed.

No RCTs have been conducted on β-blockers post-MI since the introduction of primary PCI and modern secondary prevention treatment. Several registers based observational studies with many included patients have been conducted to address the question if β-blockers as secondary prevention after acute MI still is associated with improved prognosis [5–7, 18–24]. A meta-analysis published in 2015 included 10 observational studies published between 2000 and 2014 [8]. The authors concluded that β-blocker prescription after acute MI was associated with a reduction of all-cause death during 1-year follow-up compared to patients without β-blocker prescription. There was no difference however in cardiac death, readmission for MI or heart failure [8]. Further, the study revealed that patients suffering from a NSTEMI, had LVDF or who were sub optimally treated for secondary prevention had the strongest association with better outcome when treated with a β-blocker [8].

The present study differs in several ways from the studies included in the meta-analysis [8]. The criteria for inclusion in the no-β-group (were patients < 80 years, with a type-1 MI (i.e. STEMI and NSTEMI), discharged alive without a β-blocker and with absence of AV-block II-III, which differs from studies that only included patients with STEMI and who underwent PCI [6, 7, 18, 22–24]. A study by Choo and co-workers included patients with preserved systolic function after acute MI, treated with PCI, and a study by Ellis and co-workers included patients with acute MI treated with PCI [19, 21]. A study by Chen and co-workers investigated the effect of β-blockers in elderly patients and thus included only patients > 65 years [5].

Unique to this study was that we collected information on the reason why patients were discharged without β-blocker treatment. All of these differences constitute important reasons why comparison between studies becomes difficult and may explain why the results differ. Results from the present study regarding the clinical outcome of all-cause death was not consistent with results from the meta-analysis, where both statistically unadjusted relative risk, and statistically adjusted hazard ratio for all cause death was lower in the group treated with β-blockers [8]. Regarding the study outcome of readmission for MI, the present study could show a lower incidence with β-blocker treatment, whilst the meta-analysis did not show an association of β-blocker treatment and a lower risk for MI [8]. Another possible explanation might be found among the reasons why patients were not prescribed a β-blocker, instead of the absence of a β-blocker per se, as described in Fig. 1.

In an observational study such as the present, the β- and no-β-group differ in a systematically fashion. Among the known reasons in the present study why patients were not prescribed a β-blocker were peripheral limb ischemia, patient negative to drugs and the presence of multiple comorbidities. This indicates comorbidities not found among the baseline characteristics. On the contrary, in the present study, some patients in the no-β-group were not prescribed a β-blocker since they had normal blood pressure, or a normal EF. This is in line with current treatment recommendation from ESC, that recommends β-blockers mainly to patients with reduced left ventricular function post MI [11, 25]. Since the reasons why patients were not treated with a β-blocker were not reported in the meta-analysis, one might speculate that some of these patients were instead considered too healthy for this treatment, perhaps to a larger extent than in the present study, since the Swedish quality registry provides an incentive that all patients without contraindications should be prescribed a β-blocker [26]. This might be an explanation for the different results regarding readmissions for MI.

There were some significant differences in baseline characteristics between the groups (Table 2). These included a significantly lower prevalence of EF < 50%, and a lower rate of ACE-inhibitors prescription in the no-β-group at discharge. Mean systolic blood pressure at discharge was higher in the β-group. However, the groups did not differ in risk factor profile prior to admission, mean age or final diagnosis. Both groups were to a large extent prescribed statins, ASA and ADP inhibitors.

In the present study, the main significant clinical differences between the groups, were likely associated with the absence or presence of β-blocker at discharge. Lower mean heart rate and mean systolic blood pressure at admission in the no-β group was expected since a common reason to why many patients were not prescribed a β-blocker according to the medical records was bradycardia (*n* = 54, (38%)), and to a lesser extent hypotension (*n* = 13, (9%)). At discharge, the groups did not differ in mean heart rate, and instead there was lower mean blood pressure in the β-group. An explanation might be that the β-group also had a significantly higher prescription of ACE inhibitors at discharge, which gives a more potent blood pressure treatment. The higher prevalence of heart failure in the β-group, or the higher prevalence of hypotension in the no-β-group, may explain the higher prescription rate of ACE inhibitors in the β-group. The higher rate of calcium channel blocker prescription in the no-β-group is probably as an antihypertensive and anti-ischemic drug, as an alternative to of ACE inhibitors and β-blockers.

The fact that the patient group characteristics in the present study differs from that in other observational studies on β-blockers post-MI might depend on the differences in inclusion criteria previously described. It might also depend on differences in local guidelines with regard to which patients should receive β-blockers. Additionally, these differences in patient characteristics between the two groups possibly illustrates a clinical approach that reflects the current scientific evidence, suggesting that β-blockers are more beneficial in patients with chronic heart failure, left ventricular dysfunction and larger infarcts [27].

Considering these difficulties in interpreting the results of observational registry-based studies, and the necessity to determine the place of β-blockers in contemporary acute coronary care, a clinical trial of β-blockers after MI is warranted. There is a study currently ongoing within the Riks-HIA registry, REDUCE-SWEDEHEART, where 7000 patients are going to be randomized to either treatment with β-blocker or no β-blocker, with 3500 patients in each arm [28]. (ClinicalTrials.gov identifier: NCT03278509).

### Study limitations

The intention to answer the question whether the absence of β-blocker treatment post-MI is associated with a higher rate of death and readmission to hospital for cardiovascular events, is hampered by several study limitations.

First of all, this being a retrospective observational study, the most important study limitation consists of the selection bias, since the majority of the patients were treated with or without a β-blocker after taking into account their individual clinical characteristics. Another problem with observational studies is the issue of adjusting for all possible confounders, which is not possible [29]. Important confounders not measured in this study would be other major risk factors for cardiovascular disease, such as low socioeconomic status, lack of physical activity, family history of coronary artery disease, autoimmune and inflammatory diseases [30]. In the present study, no statistical adjustment for measured confounder were done, partially since the patient groups did not differ in the measured aspects of risk factor profile and was thus considered comparable. No statistical adjustments such as propensity scores was calculated due to a small number of patients.

Moreover, the small number of patients in the present study renders a low statistical power. Statistical power is also affected by the expected effect of the treatment. In this case, to illustrate the effect of β-blocker treatment post-MI, the number needed to treat to avoid one death for long-term β-blockers have in meta-analysis of RCTs conducted in the pre-reperfusion era been 82 [31]. This indicates the need for a large number of patients in a study with this research question. On the other hand, the relatively small number of included patients enabled validation of register data and additional information from the medical records.

## Conclusion

A majority of the patients in the no-β-group had a justified absence of a β-blocker. β-blocker treatment post-MI showed a trend to fewer readmissions for MI. But as illustrated by this study, patient specific factors not visible in registry data affects whether a patient were prescribed a β-blocker post-MI or not. This complicates the interpretation of the quality index (Table 1) and will affect the quality and interpretation of studies conducted based solely on registry data, since important information is lacking to draw firm conclusions.

## Data Availability

The datasets used and/or analysed during the current study are available from the corresponding the first author and author HW on request.

## Abbreviations

MI: Myocardial infarction
RCTs: Randomized controlled trials
PCI: Percutaneous intervention
ESC: European society of cardiology
STEMI: ST-elevation myocardial infarction
NSTEMI: Non ST-elevation myocardial infarction
LVDF: Left ventricular systolic dysfunction
Riks-Hia: Swedish national registry of cardiac intensive care
CCU: Coronary care unit
ICD: International classification of disease
ACE I: Angiotensin-converting-enzyme inhibitor
ARB: Angiotensin receptor blocker
ASA: Acetylsalicylic acid
LBBB: Left bundle branch block
LMWH: Low-molecular-weight heparin
AV-block: Atrioventricular block
COPD: Chronic obstructive pulmonary disease
